# SKA1 overexpression is associated with poor prognosis in hepatocellular carcinoma

**DOI:** 10.1186/s12885-018-5119-6

**Published:** 2018-12-11

**Authors:** Yibing Chen, Jingjing Zhao, Zhihui Jiao, Weiwei Wang, Dandan Wang, Xiaohe Yu, Zhiyong Shi, Naijian Ge, Qiuzhong Pan, Jianchuan Xia, Wancheng Niu, Ruihua Zhao, Xiaofei Zhang, Wei Du

**Affiliations:** 1 Genetic and Prenatal Diagnosis Center, Department of Gynecology and Obstetrics, First Affiliated Hospital, Zhengzhou University, Zhengzhou, 450052 Henan China; 2 State Key Laboratory of Oncology in South China, Collaborative Innovation Center for Cancer Medicine, Department of Biotherapy, Sun Yat-sen University Cancer Center, Guangzhou, 510060 Guangdong China; 3 Department of Pathology, First Affiliated Hospital, Zhengzhou University, Zhengzhou, 450052 Henan China; 4 Key Laboratory for Biotech-Drugs Ministry of Health, Key Laboratory for Modern Medicine and Technology of Shandong Province, Key Laboratory for Rare & Uncommon Diseases of Shandong Province, Back and Neck Pain Hospital, Shandong Academy of Medical Sciences, Jinan, 250062 Shandong China; 5 Department of Radioactive Intervention, Eastern Hepatobiliary Surgery Hospital, Second Military Medical University, Shanghai, 200438 China; 6 Department of General Surgery, Huaihai Hospital, Xuzhou Medical University, Xuzhou, 221004 Jiangsu China; 7 Department of Medical Oncology, First Affiliated Hospital, Zhengzhou University, 1 Jianshe Road East, Zhengzhou, 450052 Henan China; 8 Department of Neurosurgery, First Affiliated Hospital, Zhengzhou University, 1 Jianshe Road East, Zhengzhou, 450052 Henan China

**Keywords:** SKA1, Hepatocellular carcinoma, Oncofetal gene, Prognosis, Gene profiling

## Abstract

**Background:**

SKA1, an important mitosis protein, has been indicated in the initiation and progression of several malignancies. However, its clinical significance in hepatocellular carcinoma (HCC) remain to be elucidated.

**Methods:**

mRNA expression of SKA1 was examined in 126 HCC and paired non-neoplastic tissues using real-time PCR and validated in The Cancer Genome Atlas (TCGA) database. SKA1 protein expression was detected using immunohistochemistry in the 126 HCC tissues and its associations with clinicopathological parameters and prognosis were analyzed. Hierarchical cluster analysis and gene set enrichment analysis (GSEA) were performed in selected Gene Expression Omnibus data sets.

**Results:**

SKA1 mRNA expression was significantly elevated in HCC tissues from both local hospital and TCGA database. Immunohistochemistry revealed that increased SKA1 expression was present in 65 of the 126 cases and was significantly associated with higher serum alpha-fetoprotein concentration, larger tumor size and higher TNM stage. Patients with positive SKA1 expression showed significantly worse overall and relapse-free survival. Multivariate Cox regression analysis revealed that SKA1 was an independent predictor of patient prognosis. Gene expression profiling analysis of public data showed that high-SKA1 expression HCC tissues had similar gene expression profiles with fetal liver tissues. Moreover, GSEA showed that genes up-regulated in high SKA1 HCC subgroup were significantly enriched in cell cycle pathway, while genes down-regulated were significantly enriched in apoptosis pathway.

**Conclusions:**

Our findings indicate that the oncofetal gene SKA1 might be involved in the progression of the HCC and could serve as a prognostic marker for HCC.

**Electronic supplementary material:**

The online version of this article (10.1186/s12885-018-5119-6) contains supplementary material, which is available to authorized users.

## Background

Hepatocellular carcinoma (HCC) is the sixth most common cancer and the third leading cause of cancer death, with 782,000 newly-diagnosed cases and 746,000 deaths in the year of 2012 worldwide [1]. Moreover, the increasing incidence of HCC makes a serious challenge to the public health. Despite current advances in the early diagnosis and treatment of HCC, the five-year survival rate of HCC remains unsatisfying. Chronic viral hepatitis, alcohol abuse, obesity and metabolic syndrome are generally considered as risk factors for HCC [2, 3]. During the pathogenesis of HCC, the expression of thousands of genes has been aberrantly changed, including the activation of oncogenes and inactivation of tumor suppressor genes [4]. Therefore, identification of novel molecular markers whose aberrant expression are involved in the initiation or progression of HCC may be helpful for the diagnosis and treatment of this disease.

Human spindle and kinetochore associated complex subunit 1 (SKA1) is a microtubule-binding protein of the outer kinetochore that is essential for stabilizing kinetochore-spindle microtubule attachment and proper chromosome segregation during mitosis [5]. Depletion of SKA1 can lead to severe defects in chromosome segregation, whereas overexpression of SKA1 results to the nucleation of interphase microtubules [6, 7]. Recent data have revealed that SKA1 is involved in the development of cancer. Li et al. have reported that upregulation of SKA1 can result in spontaneous tumorigenesis in the transgenic mouse model [8]. Moreover, overexpression of SKA1 has also been found in gastric, oral and prostate cancer, and can promote cancer cell proliferation and colony formation in these malignancies [9–11]. As one of the key factors for mitosis, SKA1 exhibits high expression level in the fetal liver with robust cell division, then diminishes gradually during development, and maintains at a very low level in the adult liver according to massive peptide sequencing data in the HUMAN PROTEOME MAP project [12]. Qin et al. have analyzed the the expression of SKA1 in 38 HCC cases and found that SKA1 expression was upregulated in HCC tissues [13]. These findings are consistent with the characteristics of some tumor oncofetal proteins, allowing us to hypothesize that SKA1 might play a role like oncofetal gene in HCC [14]. However, the clinical significance of SKA1 in HCC has not yet been investigated. In this study, we investigated the clinical value of SKA1 in 126 HCC cases and gene expression profiling in fetal liver and HCC samples with different SKA1 levels in public data. Furthermore, affected oncogenic pathways by SKA1 were identified by gene set enrichment analysis (GSEA) in HCC samples based on public data [15].

## Materials and methods

### Patients and samples

The paired tumorous and matched non-neoplastic tissues were collected from 126 HCC patients between January 2013 to December 2014 at the Eastern Hepatobiliary Surgery Hospital, Second Military Medical University in Shanghai, China. Patients who met all following criteria of eligibility were included in our study: (1) diagnosis of primary HCC identified by histopathological examination; (2) treatment with radical resection; (3) availability of complete follow-up data; (4) no preoperative anticancer treatment, such as chemotherapy, radiotherapy, immunotherapy or molecular targeted therapy; (5) no history of familial malignancy or other synchronous malignancy, and (6) no death within 3 months after operation. The disease stage was determined according to the 7th TNM staging system of the Union for International Cancer Control (UICC) and American Joint Committee on Cancer (AJCC) [16].Overall survival (OS) was defined as the time from the operation to patient death or the last follow-up. Relapse-free survival (RFS) was defined as the time elapsed from operation to the date of the recurrence or distant metastasis of HCC. This study was approved by the Ethical Committees of the Second Military Medical University and Zhegnzhou University, and written informed consent was obtained from all participants. All study procedures were carried out in accordance with the ethical standards of the Helsinki Declaration. The fresh tissues were immediately immersed in RNAlater (Ambion, Austin, TX, USA) after surgical resection and then stored at − 80 °C until experiment.

### RNA extraction and quantitative real-time PCR (qPCR)

Total RNA extraction, cDNA synthesis and quantitative real-time PCR were performed as previously described using following primer pairs: *SKA1* sense: 5’-GGTTTCACCGTGTTAGCC-3′, *SKA1* antisense: 5’-GCGTATTCAGCAGGTAGTT-3′; GAPDH sense: 5’-CTCCTCCTGTTCGACAGTCAGC-3′, GAPDH antisense: 5’-CCCAATACGACCAAATCCGTT-3′ [17]. The Ct (threshold cycle) value of each sample was calculated, and relative expression of SKA1 mRNA was normalized to the GAPDH value (2^-ΔCt^ method).

### Immunohistochemistry (IHC)

To examine the protein level of SKA1, we performed IHC analysis in the 126 paraffin-embedded HCC tissue blocks as previously described with a rabbit anti-human SKA1 polyclonal antibody (1:500, Millipore Sigma, St. Louis, MO, USA) [18]. For negative controls, adjacent sections were processed as described above except that they were incubated overnight at 4 °C in blocking solution without the primary antibody. The intensity and extent of SKA1 immunostaining were evaluated for all samples under double-blinded conditions. In brief, the percentage of positive staining was scored as 0 (0–9%), 1 (10–25%), 2 (26–50%), 3 (51–75%) or 4 (76–100%), and the intensity as 0 (no staining), 1 (weak staining), 2 (moderate staining) or 3 (dark staining). The total score was calculated as the product of intensity and extent, ranging from 0 to 12. The expression level of SKA1 was divided into negative (score 0) and positive (scores 1–12) staining.

### Public data selection and utilization

To verify SKA1 mRNA expression levels in our results, the RNA sequencing data of all matched tumor and normal samples (50 pairs) from The Cancer Genome Atlas (TCGA) were utilized (https://cancergenome.nih.gov/) [19]. The SKA1 protein levels in fetal liver and adult liver were obtained from HUMAN PROTEOME MAP web site (http://humanproteomemap.org/). To investigate the gene expression profiling in HCC with high and low SKA1 expression, hepatocyte and fetal liver samples, corresponding samples from GEO datasets (GSE6222, GSE6764, GSE9843, GSE15238, GSE18269, GSE23343, GSE29721, & GSE33606) were utilized [15]. Specifically, 6 fetal liver samples, 6 hepatocyte samples and all tumor samples were selected according to the sample information firstly, then six tumor samples with highest SKA1 level and six with lowest SKA1 level were defined as the high SKA1 HCC and low SKA1 HCC respectively.

### Gene expression profiling analysis and GSEA

The gene expression profiling in high SKA1 HCC, low SKA1 HCC, hepatocyte and fetal liver samples was analyzed by hierarchical clustering and heatmap with R and BioConductor packages. The normalized microarray data of the four groups was subtracted from the log_2_-transformed normalized data prior to clustering. Genes with no significant changes between the four groups of samples were not represented in the heatmap to show clear patterns. GSEA was performed using normalized data by GSEAv2.1tool (http://software.broadinstitute.org/gsea/index.jsp) [15]. High SKA1 HCC and fetal liver samples were defined as SKA1 high phenotype, while low SKA1 HCC and hepatocyte samples were defined as SKA1 low phenotype when did GSEA analysis.

### Statistical analysis

A paired *t*-test was used to compare the mRNA and protein expression of SKA1 in tumor and matched non-neoplastic samples. The relationship between SKA1 expression and the various clinicopathological characteristics was analyzed by the χ^2^ test. Survival curves were calculated using Kaplan-Meier method and compared by long-rank test. A Cox proportional hazard regression model was used for univariate and multivariate analyses to explore the effects of the clinicopathological variables and SKA1 expression on survival. Statistical analyses were performed using IBM SPSS Statistics 19.0 software and a *P* value < 0.05 was considered significant.

## Results

### Characteristics of patients

The clinical characteristics of the 126 HCC patients were summarized in Table 1. The population included 113 men and 13 women with a median age of 53 years (range, 18–79 years). In detail, 113 patients were hepatitis B virus surface antigen (HBsAg)-positive, 74 patients had higher serum AFP concentration, 63 patients had maximal diameter of tumor ≤5 cm and 97 patients had grade I or II tumors.

**Table 1:** Association of SKA1 expression in tumor tissues with characteristics of HCC patients

Variables	*n*	SKA1 expression		*P* value
		Positive Negative		
All cases	126	65	61	
Gender				0.317
Female	13	5	8	
Male	113	60	53	
Age (year)				0.457
< 53	60	33	27	
≥ 53	66	32	34	
Tumor size (cm)				0.002
< 5	63	24	39	
≥ 5	63	41	22	
Differentiation				0.155
Well to moderate	34	14	20	
Poor	92	51	41	
HBsAg				0.272
Positive	114	57	57	
Negative	12	8	4	
Serum AFP (μg/L)				0.035
≥ 200	74	44	30	
< 200	52	21	31	
TNM stage				0.009
I + II	92	41	51	
III + IV	34	24	10	

*AFP*, α-fetoprotein; *HBsAg*, hepatitis B virus surface antigen; *HCC*, hepatocellular carcinoma

### SKA1 mRNA expression was increased in HCC tissues

**Fig. 1:**
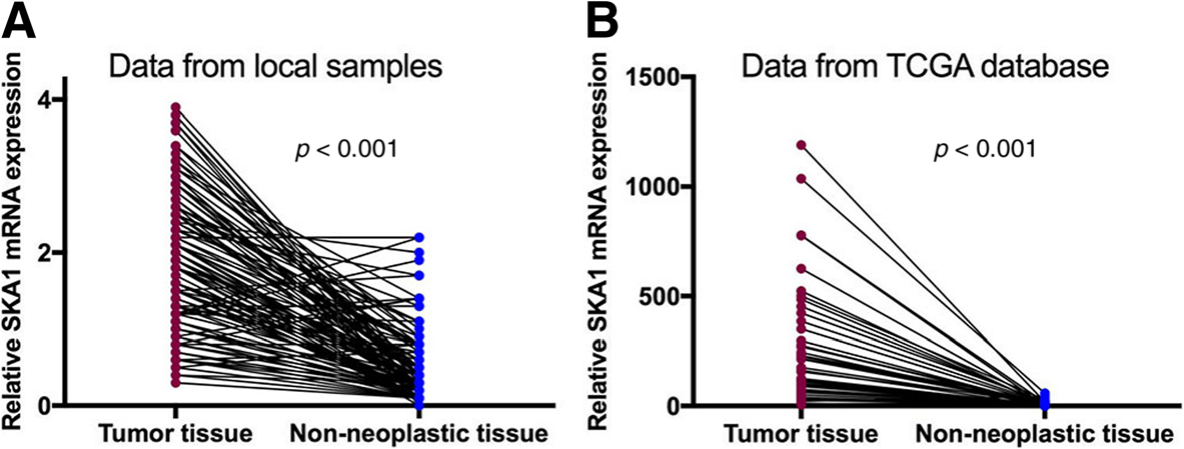
Elevated SKA1 mRNA level in HCC tissues. **a**, Relative mRNA expression levels in 126 paired HCC and pericancer tissues determined by real-time PCR in our study. **b**, mRNA expressions determined by RNA profiling in TCGA database

Real-time quantitative PCR was performed in HCC tissues and the matched non-neoplastic tissues to determine their SKA1 mRNA levels. The results indicated that SKA1 mRNA expression was significantly higher in tumorous tissues than that in non-neoplastic tissues (*P* < 0.001, Fig. 1a). Furthermore, our results were also verified by RNA sequencing data (RNASeqV2) from TCGA database which showed significantly higher SKA1 expression in tumorous tissues (*P* < 0.001, Fig. 1b and Additional file 1).

### IHC analysis of SKA1 expression in HCC and its relationship with the clinicopathological parameters

**Fig. 2:**
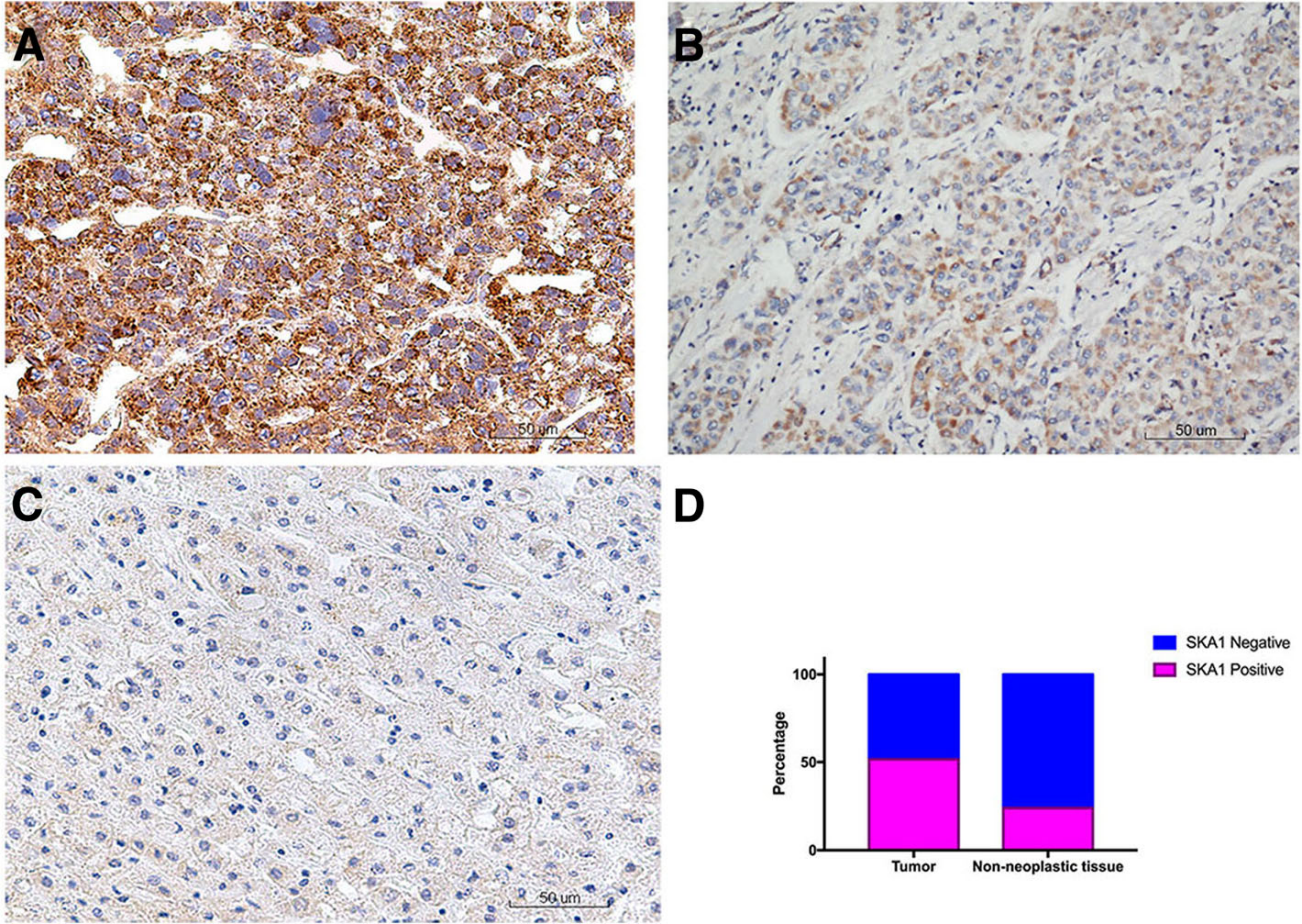
SKA1 expression in HCC tissues by IHC staining. **a**, intensive SKA1 staining in tumor tissues. **b**: moderate SKA1 staining in tumor staining. **c**, negative SKA1 staining in non-neoplastic liver tissues. **d**, proportion of different SKA1 staining in tumor and non-neoplastic tissues

To validate the findings in mRNA expression, we performed IHC analysis of SKA1 expression in the 126 HCC tissues. We observed differential expression of SKA1 in matched tissues, with more SKA1-expressing cells in the tumorous tissues than in the matched non-neoplastic tissues. Overall, 65 of 126 (51.6%) cases showed positive SKA1 immunostaining in tumorous tissues, whereas 96 (76.2%) cases showed negative SKA1 immunostaining in non-neoplastic tissues (Fig. 2). χ^2^ test showed that positive SKA1 expression was significantly correlated with higher serum AFP (*P* = 0.035), larger tumor size (*P* = 0.002) and late TNM stage (*P* = 0.009), but not with gender, age, differentiation or HBsAg (Table 1).

### SKA1 expression and clinical outcome

**Fig. 3:**
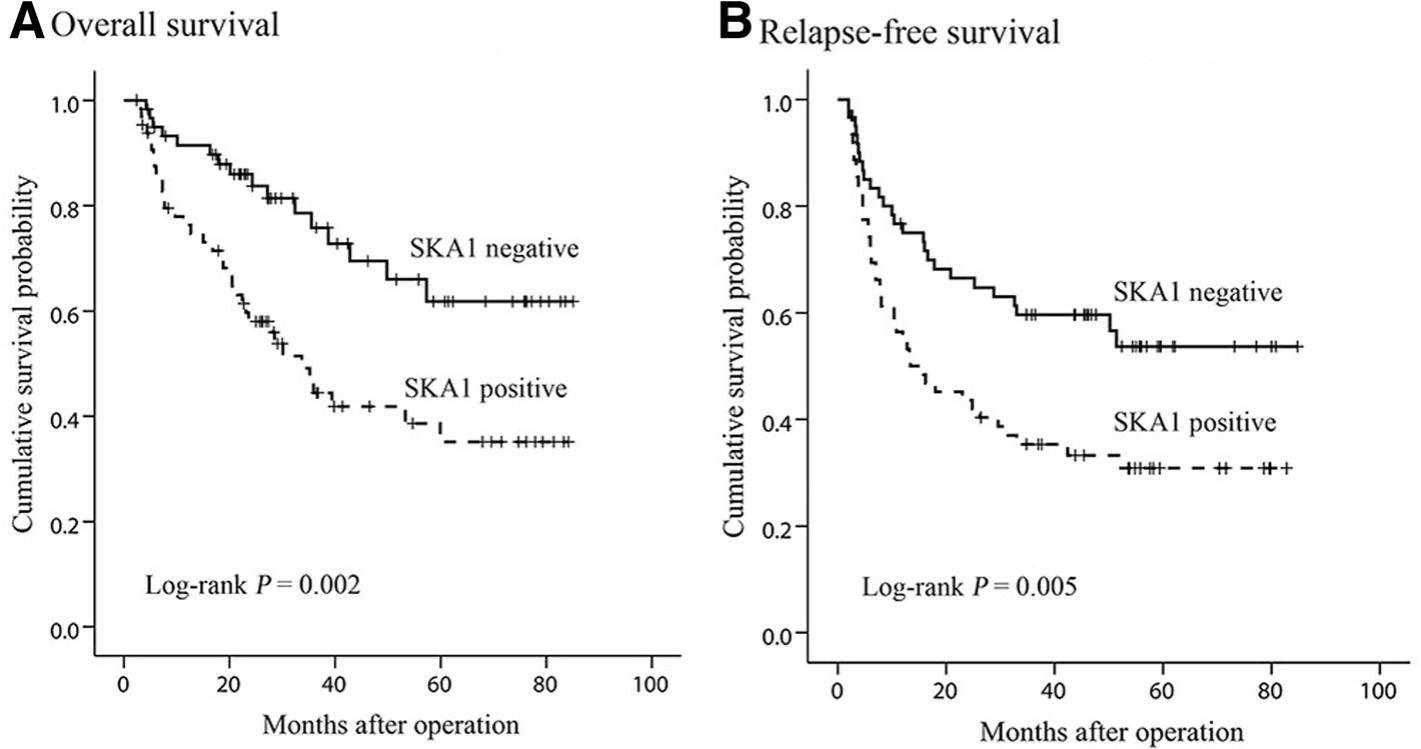
Kaplan-Meier survival curves of HCC patients by SKA expression in tumor tissues. **a**, OS curves stratified by SKA1 expression. **b**, RFS curves stratified by SKA1 expression

Kaplan-Meier curve analysis showed that patients with positive SKA1 expression had significantly poorer OS (*P* = 0.002) and RFS (*P* = 0.005) than those with negative expression (Fig. 3). Univariate Cox regression analysis showed that both death and relapse risk of HCC patients were increased with higher serum AFP, larger tumor size, late TNM stage and positive SKA1 expression (Table 2). Multivariate analysis confirmed that SKA1 expression was an independent prognostic factor of OS (HR, 2.133; 95% CI, 1.145–3.974; *P* = 0.017) (Table 2). Furthermore, SKA1 expression was also an independent prognostic factor of RFS (HR, 1.750; 95% CI, 1.064–2.878; *P* = 0.028) (Table 2).

**Table 2:** Cox regression analysis of prognostic factors for HCC patients

Variables	OS				RFS			
	Univariate analysis		Multivariate analysis		Univariate analysis		Multivariate analysis	
	HR (95% CI)	*P*	HR (95% CI)	*P*	HR (95% CI)	*P*	HR (95% CI)	*P*
Gender(male/female)	1.355 (0.537–3.416)	0.520			1.621 (0.652–4.031)	0.299		
Age (> 53/≤53 years)	1.446 (0.827–2.527)	0.195			1.990 (1.970–2.011)	0.454		
Tumor size (> 5/≤5 cm)	2.275 (1.296–3.994)	0.004			2.603 (1.583–4.281)	0.001		
Differentiation (poor/well to moderate)	1.835 (0.954–3.530)	0.069			1.213 (0.721–2.040)	0.466		
HBsAg (positive/negative)	1.323 (0.526–3.328)	0.552			1.147 (0.524–2.509)	0.731		
AFP (≥200/< 200 μg/L)	1.000 (1.081–3.281)	0.025	1.395 (0.782–2.489)	0.260	1.912 (1.183–3.090)	0.008	1.449 (0.857–2.450)	0.167
TNM stage (III + IV/I + II)	1.516 (1.156–1.987)	0.003	1.962 (1.196–3.684)	0.031	1.688 (1.337–3.131)	0.001	1.472 (1.131–1.916)	0.004
SKA1 expression (positive/negative)	2.497 (1.380–4.516)	0.002	2.133 (1.145–3.974)	0.017	1.974 (1.208–3.224)	0.007	1.750 (1.064–2.878)	0.028

*AFP*, α-fetoprotein; *CI*, confidence interval, *HBsAg*; hepatitis B virus surface antigen; *HCC*, hepatocellular carcinoma; *HR*, hazard ratio; *OS*, overall survival; *RFS*, relapse-free survival

### High SAK1 level was associated with fetal liver gene profiling and activated cell cycle pathway

**Fig. 4:**
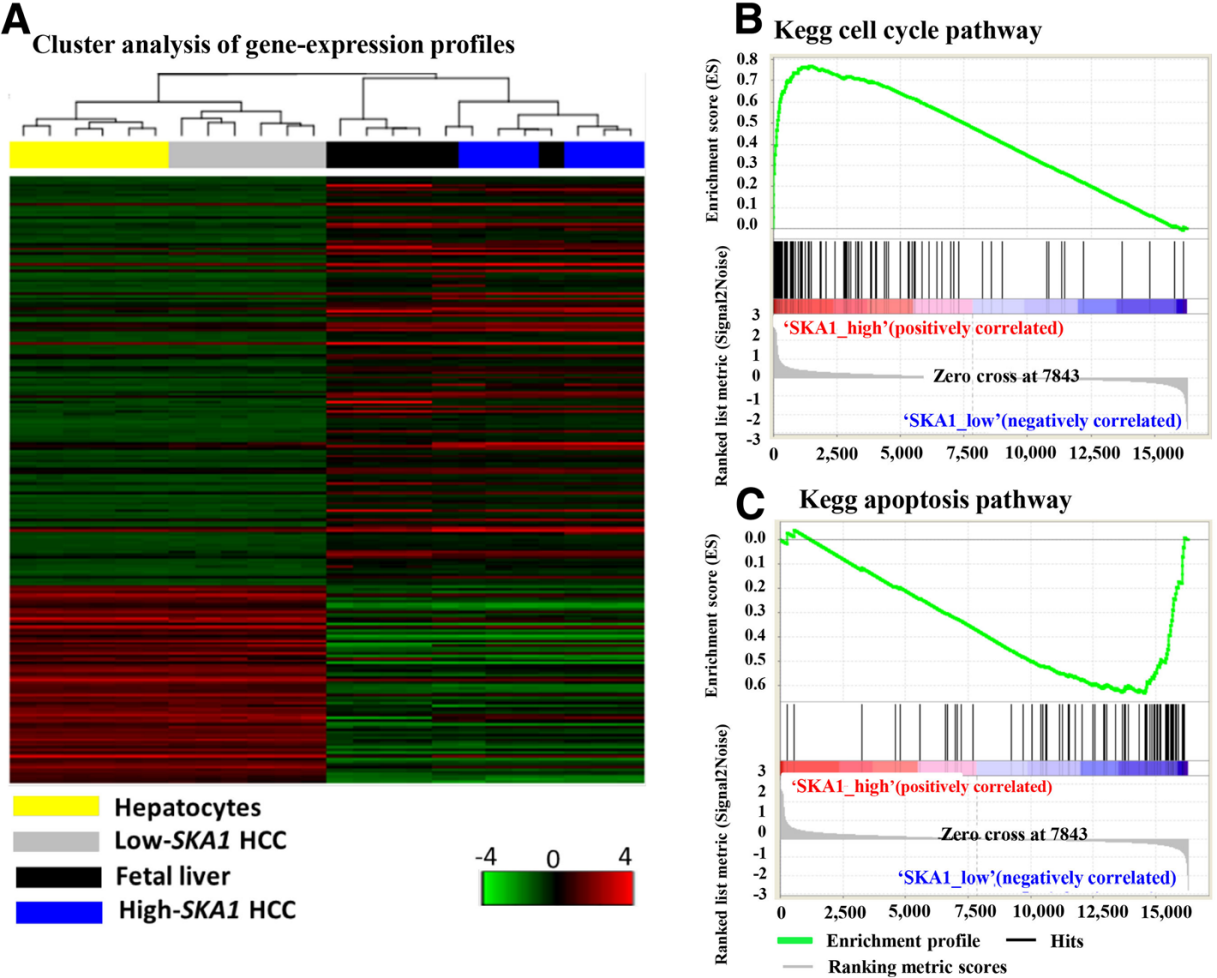
Gene profiling analysis of four types of sample including high SKA1, low SKA1, fetal liver and hepatocytes (adult liver) based on microarray data. The dendrogram and heatmap (**a)** show the hierarchical cluster analysis of gene-expression data from human specimens of hepatocytes, fetal liver and HCC tissues. Columns represent individual samples, and rows represent individual genes. Each cell in the matrix represents the expression level of a gene in an individual sample. The scale bar indicates the level of expression, red indicates a high level of expression, and green a low level of expression. GSEA analysis showed that genes upregulated in high SKA1 HCC enriched in the cell cycle pathway (**b**), while genes down-regulated in the high SKA1 HCC were enriched in the apoptosis pathway (**c**). NES denotes normalized enrichment score in gene set enrichment analysis. The ranked list metric was generated by calculating the signal-to-noise ratio which is based on the difference of means scaled according to the standard deviation. The larger the signal-to-noise ratio, the more distinct the gene expression is in each phenotype and the more the gene acts as a “class marker.” The bar codes stand for genes in the pathway. The Broad Institute Gene Set Enrichment Analysis website (http://software.broadinstitute.org/gsea/index.jsp) provides detailed information about the computational method

To investigate whether high SKA1 HCC shared a gene expression pattern with fetal liver, we performed hierarchical cluster analysis using gene expression microarray data of four groups with different SKA1 levels (high SKA1 HCC, low SKA1 HCC, hepatocyte and fetal liver) in extracted from GEO database. Our results showed that high SKA1 HCC clustered tightly with fetal liver, whereas the low SKA1 HCC clustered with hepatocytes (Fig. 4a). We further performed GSEA to investigate the enrichment of oncogenic pathways in high SKA1 HCC samples. Our results indicated that genes up-regulated in high SKA1 HCC subgroup were significantly enriched in cell cycle pathway (Fig. 4b), while genes down-regulated were significantly enriched in apoptosis pathway (Fig. 4c). All these findings indicated that cancer cells have similar gene expression profiling with fetal liver cells in HCC patients with high SKA1 level, and this type of gene profiling could promote cell proliferation by promoting cell cycle progression and reducing cell apoptosis.

## Discussion

HCC is one of the deadliest cancers in the world, and has a dismal prognosis despite improved early diagnosis and combined therapy [1]. Elucidating the molecular mechanisms underlying HCC would be helpful to the development of novel therapeutics [20]. This study showed the SKA1 expression was significantly increased in HCC tissues compared with matched non-neoplastic tissues. Gain of SKA1 expression was associated with higher serum AFP concentration, larger tumor size and higher TNM stage. Patients with high SKA1 level tend to have a poor prognosis probably due to the fetal liver cell-like gene expression profiles in cancer cells [21], and such gene profiling was associated with cell cycle and apoptosis pathways that may promote cancer cell proliferation.

Emerging evidence has demonstrated SKA1 as a candidate oncofetal protein that is involved in the development of a range of cancer types. SKA1 overexpression has been observed in gastric cancer, oral adenosquamous carcinoma, prostate cancer, papillary thyroid carcinoma, non-small cell lung cancer, and salivary adenoid cystic carcinoma [9–11, 22–24]. In consistence with these findings, we found that SKA1 expression was elevated at the both mRNA and protein levels in the majority of HCC tumorous tissues, which was further confirmed by our TCGA data analysis. Similarly, a previous study by Qin et al. has also shown that SKA1 is upregulated in HCC tissues [13]. However, their sample size to not large enough to explore the clinical significance of SKA1 expression in HCC. Collectively, these data on the expression of SKA1 in HCC imply that SKA1 reactivation might be an important event during cancer development.

SKA1 is a key component of SKA1 complex that is essential for stabilizing kinetochore–spindle microtubule attachment during mitosis [25]. Loss-of-function mutations in SKA1 result in chromosome congression failure and subsequent cell death, suggesting the important roles in cell survival and proliferation of SKA1 [26]. In addition, SKA1 is also abundant at centrosomes and indispensable for centriole biogenesis [8]. Moreover, centrosome amplification has been frequently observed in various solid tumors and is often correlated with poor prognosis of patients [27, 28]. Therefore, it is reasonable that SKA1 was highly expressed in robustly-dividing cells, such as fetal liver and HCC. This specific temporal expression pattern indicates that SKA1 might act as a candidate oncofetal gene in HCC. Previous studies have demonstrated that SKA1 upregulation is associated with higher tumor stage, aggressive phenotype and poor prognosis of patients [22–24, 29]. In line with these findings, we confirmed that SKA1 overexpression was significantly associated with larger tumor size, higher TNM stage, serum AFP level and poor prognosis of HCC patients, suggesting that SKA1 may promote the growth and progression of cancer.

It is considered that SKA complex plays important roles in inhibiting spindle checkpoint signaling, which promote anaphase onset and thus cell cycle progression [30]. Indeed, previous studies have shown that SKA1 upregulation enhances cell proliferation by promoting cell cycle progression in prostate cancer, gastric, bladder, and oral cancers [9–11, 31, 32], whereas depletion of SKA1 expression using RNA interference causes cell cycle arrest in cancer cells possibly by inhibiting the expression of cyclin D1 and CKD4 [31, 32]. Moreover, SKA1 silencing also induces cell apoptosis in prostate cancer and oral adenosquamous carcinoma [10, 11], suggesting SKA1 may be involved in the apoptotic resistance in cancer. In this study, our GSEA analysis demonstrated that high SKA1 overexpression was correlated with high expression of cell cycle-related gene sets and low expression of proapoptotic gene sets. This was confirmed by the findings of Qin et al., which have shown that lentivirus-mediated siRNA against SKA1 inhibits HCC cell proliferation by inducing cell cycle arrest in the G0/G1 phase while promoting apoptosis [13]. Besides, SKA1 upregulation is significantly associated with the metastasis of NSCLC [23], and cell studies have revealed that knockdown of SKA1 expression by siRNA represses the invasion and metastasis of a range of cancer types, including renal cell carcinoma, salivary adenoid cystic carcinoma, prostate cancer, glioblastoma and NSCLC [23, 29, 31, 33]. In addition, SKA1 has shown to be involved in the chemoresistance of cancer cells [23, 34]. These data collectively suggest that SKA1 may play multifaceted roles in the initiation, progression and chemosensitivity of cancer. Although several studies have shown that AKT and ERK signaling mediate the oncogenic functions of SKA1 [23, 32], the detailed mechanisms by which SKA1 exerts these effects remain unclear and thus warrant further investigations.

The reason why SKA1 expression is elevated in cancer remains unknown. Previous TCGA data analysis has shown that SKA1 mutations or copy number variations occur at a very low frequency in various cancer types [8]. Arai et al. have reported that SKA1 is the target gene of miR-10a-5p, an antitumor microRNA that is downregulated in renal cell carcinoma [29]. Further studies are needed to elucidate the molecular mechanisms by which SKA1 are dysregulated in cancer, including HCC.

## Conclusions

Our study shows that the SKA1 is an oncofetal gene and plays an important role in the cell proliferation in HCC. It can be a reasonable biomarker and prognostic factor in HCC since high level SKA1 correlates with a worse clinical outcome. To our best knowledge, the data generated in this study represent the first report correlating the presence of SKA1 with clinicopathological characteristics as well as with the survival of HCC patients.

## Additional file

Additional file 1: Expression data of SKA1 in matched HCC and non-tumor liver tissues from TCGA database. (XLS 61 kb)

## Abbreviations

AFP: Alpha-fetoprotein
AJCC: American Joint Committee on Cancer
AKT: Protein kinase B
CKD4: Cyclin-dependent protein kinase 4
ERK: Extracellular regulated protein kinases
GEO: Gene Expression Omnibus
GSEA: Gene set enrichment analysis
HCC: Hepatocellular carcinoma
IHC: Immunohistochemistry
NSCLC: Non-small-cell lung cancer
OS: Overall survival
PCR: Polymerase Chain Reaction
RFS: Relapse-free survival
SKA1: Spindle and kinetochore associated complex subunit 1
TCGA: The Cancer Genome Atlas database
UICC: Union for International Cancer Control
